# Resensitizing tigecycline- and colistin-resistant *Escherichia coli* using an engineered conjugative CRISPR/Cas9 system

**DOI:** 10.1128/spectrum.03884-23

**Published:** 2024-02-22

**Authors:** Haijie Zhang, Bo Chen, Zeyu Wang, Kai Peng, Yuan Liu, Zhiqiang Wang

**Affiliations:** 1 Joint International Research Laboratory of Agriculture and Agri-Product Safety of Ministry of Education of China, Yangzhou University, Yangzhou, China; 2 College of Veterinary Medicine, Yangzhou University, Yangzhou, China; 3 Jiangsu Co-innovation Center for Prevention and Control of Important Animal Infectious Diseases and Zoonoses, Yangzhou University, Yangzhou, China; 4 Institute of Comparative Medicine, Yangzhou University, Yangzhou, China; University of Arkansas for Medical Sciences, USA

**Keywords:** antibiotic resistance, CRISPR, *mcr-1*, resensitization, *tet*(X)

## Abstract

Tigecycline and colistin were referred to as the “last resort” antibiotics in defending against carbapenem-resistant, Gram-negative bacterial infections, and are currently widely used in clinical treatment. However, the emergence and prevalence of plasmid-mediated *tet*(X4) and *mcr-1* genes pose a serious threat to the therapeutic application of tigecycline and colistin, respectively. In this research, a tigecycline- and colistin-resistant bacteria resensitization system was developed based on efficient and specific DNA damage caused by Clustered Regularly Interspaced Short Palindromic Repeats (CRISPR)-Associated Protein 9 (Cas9) nucleases. A conjugation method was used to deliver the resensitization system, which harbors two single-guide RNAs targeting *tet*(X4) and *mcr-1* genes and constitutively expressed Cas9. The conjugation efficiency was nearly 100% after conjugation condition optimization *in vitro*, and the resensitivity efficiency for clinical isolates was over 90%. In addition, when performing resensitization *in vivo*, the resistance marker was replaced with a glutamate-based, chromosomal, plasmid-balanced lethal system to prevent the introduction of additional resistance genes in clinical settings, making this strategy a therapeutic approach to combat the *in vivo* spread of antibiotic resistance genes (ARGs) among bacterial pathogens. As a proof of concept, this resensitive system can significantly decrease the counts of tigecycline- and colistin-resistant bacteria to 1% *in vivo*. Our study demonstrates the efficacy and adaptability of CRISPR-Cas systems as powerful and programmable antimicrobials in resensitizing *tet*(X4)- and *mcr-1-*mediated, tigecycline- and colistin-resistant strains, and opens up new pathways for the development of CRISPR-based tools for selective bacterial pathogen elimination and precise microbiome composition change.

**IMPORTANCE:**

The emergence of plasmid-encoded *tet*(X4) and *mcr-1* isolated from human and animal sources has affected the treatment of tigecycline and colistin, and has posed a significant threat to public health. Tigecycline and colistin are considered as the “last line of defense” for the treatment of multidrug-resistant (MDR) Gram-negative bacterial infections, so there is an urgent need to find a method that can resensitize *tet*(X4)-mediated tigecycline-resistant and *mcr-1*-mediated colistin-resistant bacteria. In this study, we developed a glutamate-based, chromosomal, plasmid-balanced lethal conjugative CRISPR/Cas9 system, which can simultaneously resensitize *tet*(X4)-mediated tigecycline-resistant and *mcr-1*-mediated colistin-resistant *Escherichia coli*. The counts of tigecycline- and colistin-resistant bacteria decreased to 1% *in vivo* after the resensitization system was administered. This study opens up new pathways for the development of CRISPR-based tools for selective bacterial pathogen elimination and precise microbiome composition change.

## INTRODUCTION

The discovery of antibiotics is the greatest medical breakthrough of the 20th century (1). However, due to the widespread use or even misuse of antibiotics in recent years, the problem of bacterial resistance has worsened, with rising infections and isolations of resistant bacteria and the generation of novel resistance genes and mechanisms. In recent years, the number of multidrug-resistant (MDR) Gram-negative bacteria, especially carbapenem-resistant *Enterobacteriaceae* and *Fusobacterium*, has increased rapidly, making carbapenems less effective (2). As a result, colistin and tigecycline, referred to by the World Health Organization (WHO) as the “last resort” in defending against carbapenem-resistant Gram-negative bacterial infections, are currently widely used in clinical treatment (3). However, since the emergence of plasmid-mediated, colistin resistance genes (*mcr-1*) in 2015 (4) and high-level tigecycline resistance genes [*tet*(X3) and *tet*(X4)] in 2019 (5, 6), the widespread *mcr-1* and *tet*(X4) have severely compromised the use of tigecycline and colistin in clinical treatment. Furthermore, an animal-origin *Escherichia coli* isolate with the coexistence of *tet*(X4), *mcr-1*, and *bla*_NDM-5_ (7), and an *Acinetobacter baumannii* isolate from chicken carrying a plasmid-borne *tet*(X6) with *bla*_OXA-58_ and *bla*_NDM-1_ genes (8) are more likely to develop a superbug that is virtually completely resistant to all antibiotics. Since the production of new antibiotics is declining (9), restoring the susceptibility of drug-resistant bacteria has become one of the methods to overcome antibiotic resistance (10).

Clustered Regularly Interspaced Short Palindromic Repeats (CRISPR), together with CRISPR-associated (Cas) proteins, form the adaptive immune system of bacteria and archaea, providing prokaryotes with adaptive immunity against invaders (11). The type II CRISPR system of *Streptococcus pyogenes* comprises only Cas9 and two RNA (12). Jinek et al.(13) simplified the CRISPR-Cas9 system by maturing crRNA and tracrRNA to form single-guide RNAs (sgRNAs) that direct the Cas9 protein to introduce double-stranded breaks (DSB) in target DNA .

Since drug-resistant genes are mainly transmitted horizontally via plasmids, methods to treat plasmids by designing sgRNA-targeted plasmid replicons have been widely used (14). However, owing to the wide variety of replicons in natural plasmids, it is challenging to determine which plasmids contain the drug resistance genes without sequencing. In contrast, it is simpler and more efficient to target antibiotic resistance genes (ARGs) directly. In previous research, several groups have eliminated *mcr-1*-harboring plasmids in clinical *E. coli* isolates by designing sgRNA-targeted *mcr-1* genes *in vitro* (15, 16). However, only approximately 10% of resistant bacteria have become resensitive to colistin, and the efficiency has not been validated *in vivo*, which limits the guidance for clinical practice. To our knowledge, no research has been conducted to simultaneously target the *tet*(X4) and *mcr-1* resistance genes *in vivo* to resensitize tigecycline- and colistin-resistant isolates through conjugation. Given the predominance of tigecycline and colistin in the treatment of MDR Gram-negative bacterial infections, particularly carbapenem-resistant *Enterobacteriaceae*, we developed a resensitization method that can simultaneously restore susceptibility to enterobacteria with *tet*(X4) and *mcr-1* variants. A host-independent conjugative plasmid harboring the engineered CRISPR/Cas9 system with different sgRNAs targeting *tet*(X4) and *mcr-1* variants was created in this study to limit the introduction of additional resistance genes into the resensitized bacteria *in vivo*. This strategy may provide an innovative approach to combating the ever-increasing spread of antibiotic resistance genes among bacterial pathogens.

## RESULTS

### CRISPR-Cas9 system results in resensitization

**Fig 1:**
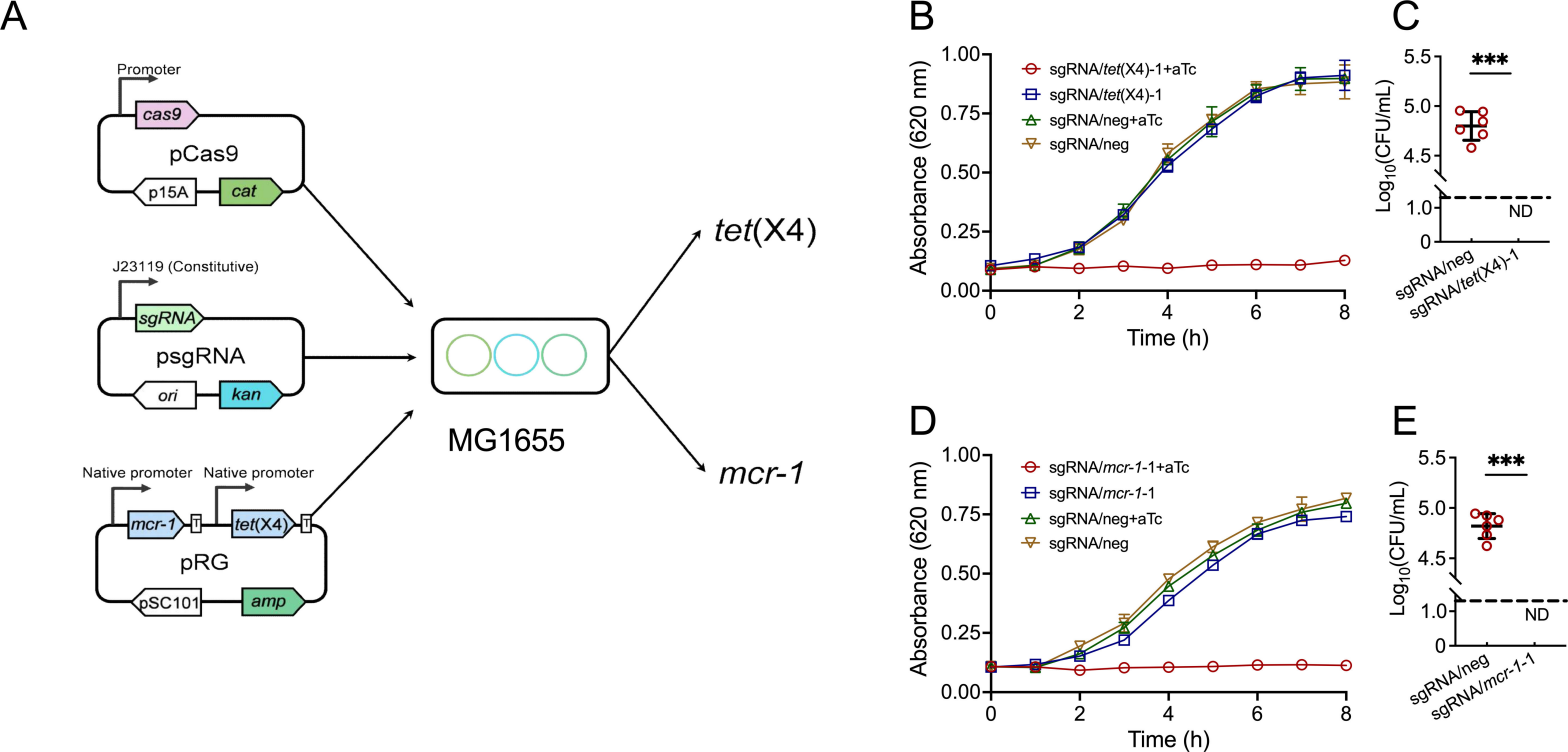
Engineered CRISPR-Cas9 system resensitizes *tet*(X4)- and *mcr-1*-carrying tigecycline- and colistin-resistant *E. coli*. (**A**) A schematic map of plasmid in CRISPR/Cas9 system. The *cas9* gene was under the control of the constitutive promoter J23110 or aTc-induced *tet*A promoter. (**B and D**) The growth curve of MG1655 transformed with pRG, pCas/Con, and psgRNA carrying single-guide RNA (sgRNA)/*tet*(X4)-1, sgRNA/*mcr-1*-1, or sgRNA/neg with or without 1µM aTc treatment in Luria–Bertani (LB) broth supplied with ampicillin (100µg/mL), chloramphenicol (25µg/mL), and kanamycin (50µg/mL). (**C and E**) The number of surviving colonies on LB plate supplied with ampicillin, chloramphenicol, and kanamycin after transformation with pRG, pCas/Ind, and psgRNA carrying single-guide RNA (sgRNA)/*tet*(X4)-1, sgRNA/*mcr-1*-1, or sgRNA/neg. Dashed lines indicate detection limitations (20CFU/mL). ND indicates that no clones have been observed on the plates. Data are shown as mean ± SD,and one‐way analysis of variance (ANOVA) was used to evaluate the statistical significance (***, P < 0.001).

Three different sgRNAs targeting the *mcr-1* or *tet*(X4) genes were designed using the CHOPCHOP tool and were cloned into psgRNA plasmid to verify the effectiveness of the CRISPR-Cas9 system in resensitizing the *mcr-1*- and *tet*(X4)-mediated colistin or tigecycline resistance bacteria. The two components of the CRISPR-Cas9 system, Cas9 driven by anhydrotetracycline (aTc)-induced promoter (pCas/Ind) and psgRNA plasmid harboring different sgRNAs driven by the constitutive promoter J23119, and the *mcr-1*/*tet*(X4) dual expression plasmid (pRG) were co-transformed into DH5α (Fig. 1A). The growth test results are presented in Fig. S1A and B. The DH5α transformed with psgRNA/neg can grow normally with aTc inducer. However, upon exposure to 1µM aTc, the DH5α containing all three sgRNA/*mcr-1* and sgRNA/*tet*(X4)-1/2 constructs exhibited the inability for growth in Luria–Bertani (LB) medium supplemented with pRG-resistant antibiotics. Although the bacteria exhibited the ability to proliferate in the presence of sgRNA/*tet*(X4)-3 substance, the rate of growth was notably reduced in comparison to the sgRNA/neg group. Then, the aTc-induced *tet*A promoter upstream of Cas9 was exchanged with the constitutive promoter J23110 (17) in pCas/Con, and the plasmids’ co-transformation efficiency was determined through transformation assays. As presented in Fig. S1C and D, the pCas/Con, psgRNA/neg, and pRG co-transformation efficiency is over 10^4^CFU/100ng DNA, while there were no detectable clones on the LB plates supplied with ampicillin when co-transformed with sgRNA/*mcr-1*-1/2/3 or sgRNA/*tet*(X4)-1/3. As the DH5α strain contains plenty of genetic mutations that affect the genetic behaviors and the physiology of the cells, the sgRNA/*mcr-1*-1 or sgRNA/*tet*(X4)-1 with the highest resensitization effectiveness was chosen for further testing in *E. coli* MG1655. As depicted in Fig. 1B through E, there was a notable decrease in the growth rate and number of transformants observed in the sgRNA/*mcr-1*-1 or sgRNA/*tet*(X4)-1 constructs when compared to the sgRNA/neg group. These findings suggested that the constitutively expressed Cas9 combined with sgRNA/*mcr-1* or sgRNA/*tet*(X4) can effectively introduce double-strand break in *mcr-1* and *tet*(X4) genes, which causes the loss of the plasmid, making it impossible for bacteria to survive in plasmid-resistant antibiotics and achieve the effect of resensitization.

### CRISPR/Cas system can deliver through conjugation

Bacterial conjugation is the primary mode of horizontal gene transfer employed by bacteria to acquire foreign DNA. To develop a resensitive system that can be transferred to target bacteria through conjugation, pTra/Ind was constructed carrying P15A replication origin, chloramphenicol-selected marker, an origin of transfer (oriT) for conjugation, aTc-induced Cas9, and sgRNA/neg. pTra/Ind was transformed into *E. coli* S17-1 as the donor strain, and rifampicin-resistant EC600 carrying pRG was used as the recipient strain. The conjugation frequency was performed at a ratio of 1:4 for donors to recipients under different conditions involving different mating times, pH values, and mating temperatures. As presented in Fig. S2A through C, the EC600 conjugated with pTra/Ind was detected after 2 h of conjugation, and the conjugation frequency peaked after 4 h and was maintained until 12 h after mixing. Moreover, the conjugation frequency was maintained at its maximum when the pH value was 5–8 and the temperature exceeded 30°C. The ratio of donor to recipient strain also affected the conjugation frequency. The conjugation frequency increased dramatically when the number of donor strains in the mixture remained constant; however, the percentage of recipient strains was reduced (Fig. S2D). The conjugation frequency was close to 100% when the ratio was less than 1:0.01. This means that almost all the recipient bacteria accepted the plasmids. These findings indicate that the efficiency of the conjugative CRISPR/Cas system can be maintained in different environments and has reached its peak when the donor/recipient ratio increased. The results indicate that the effectiveness of the conjugative CRISPR/Cas system remains consistent with the optimal efficiency presented at higher donor/recipient ratios.

### Resensitization using the CRISPR/Cas system delivered through conjugation

**Fig 2:**
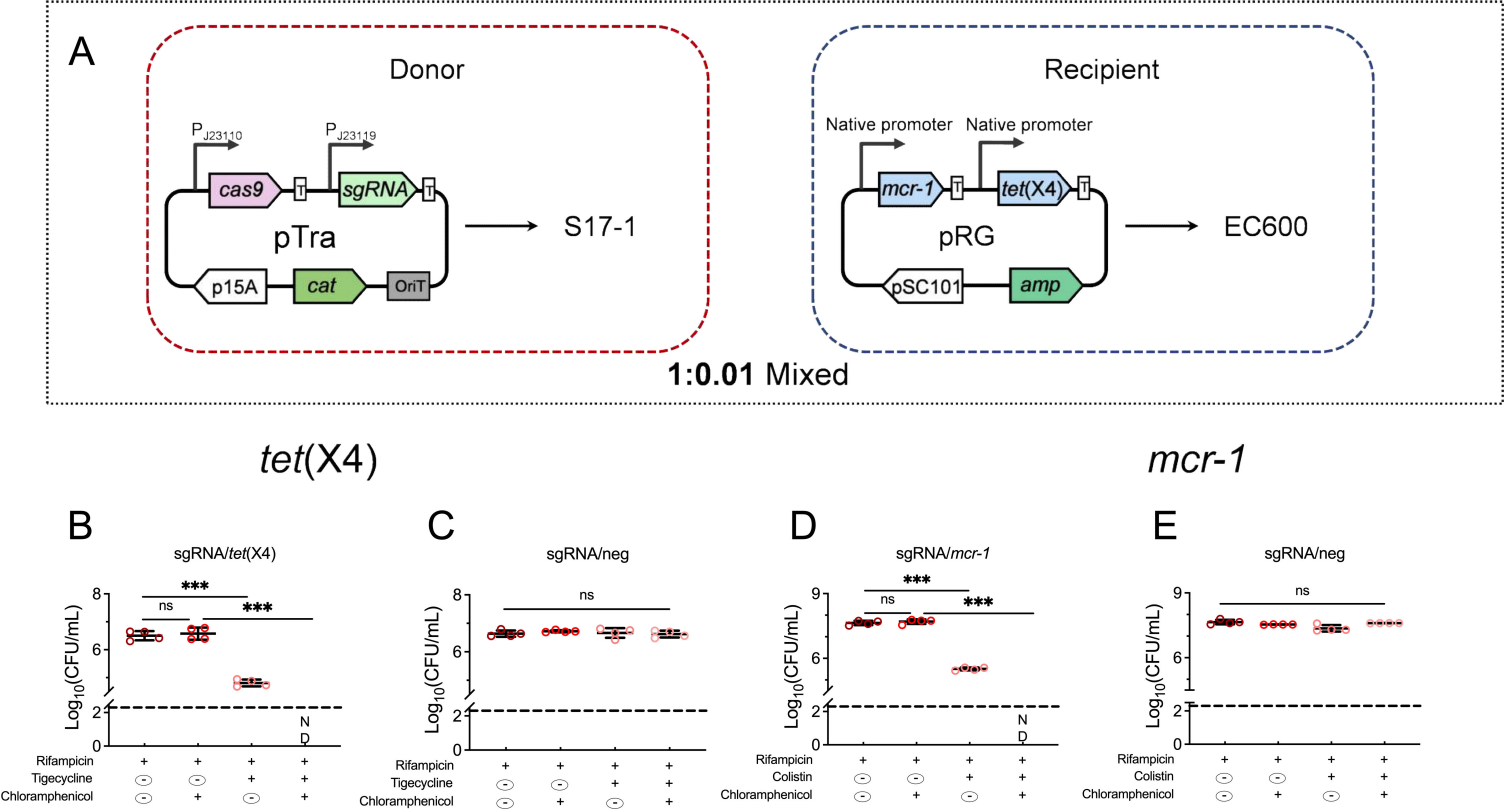
CRISPR-Cas9 system can be delivered into bacteria harboring target sequences by conjugation. (**A**) Schematic map of conjugative resensitization system. The CRISPR/Cas9 system harboring *cas9* and sgRNA under the control of constitutive promoter proD and P_J23119_ was transformed into S17-1 and was set as the donor strain. Rifampicin-resistant *E. coli* EC600 carrying *tet(X4*) and *mcr-1* was set as the recipient strain. (B to E) Conjugative CRISPR/Cas9 system harboring sgRNAs/*tet*(X4) or sgRNA/*mcr-1* cause specific clearance of plasmid pRG. *E. coli* S17-1 donor cells containing pTra/*tet*(X4) or pTra/*mcr-1* were mated with *E. coli* EC600 recipient cells carrying pRG at a donor-recipient ratio of 1:0.01 for 12h. The number of surviving colonies on the Luria–Bertani (LB) plate supplied with different antibiotics was calculated. Dashed lines indicate detection limitations (200CFU/mL). ND indicates that no clones have been observed on the plates. Data are shown as mean ± SD,and one‐way ANOVA was used to evaluate the statistical significance (ns, not significant; *** *P* < 0.001).

To further characterize the efficiency of the CRISPR/Cas system in resensitizing *tet*(X4)-mediated tigecycline resistance strains, S17-1 transformed with pTra/*tet*(X4)-1 or pTra/*mcr-1*-1 was designated as the donor, and EC600 transformed with pRG as the recipient (Fig. S3A). Following conjugation, the transconjugant and recipient were calculated on plates containing specific antibiotics. It is worth noting that tigecycline and colistin were used to screen the resistant strains, and chloramphenicol was used to confirm the presence of CRISPR/Cas system. When the donor-to-recipient ratio was 1:1, we observed that the count of total recipients, EC600, was 4.9 × 10^8^; however, the total transconjugants, EC600 with pTra/*tet*(X4)-1, was only 1.4 × 10^7^, which is consistent with the conjugation efficiency mentioned above (2.2%) (Fig. S3B). The number of tigecycline-resistant bacteria remains almost constant with that of EC600, and the resensitization efficiency is less than 20%. To obtain a maximum resensitization efficiency, a donor-recipient bacteria ratio from 1:0.01 to 1:0.0001 was tested again (Fig. S3). When the donor-to-recipient ratio was 1:0.01 (Fig. 2A through C), there was no significant difference between the recipients and the transconjugants, indicating that almost every recipient isolate received the resensitized system and that the resensitization efficiency was over 98%. Furthermore, there was no detectable co-existence of pRG and pTra/*tet*(X4)-1 observed in EC600, which indicates that the escape rate remained relatively low as the donor-to-recipient ratio increased. A similar resensitization efficiency was observed when using S17-1/pTra/*mcr-1*-1 as the donor strain (Fig. 2D and E; Fig. S3). When the donor-to-recipient ratio was 1:0.01 and 1:0.001, there were no significant differences in counts between the recipients and the transconjugants. Similarly, there were no detectable co-existence of pRG and pTra/*mcr-1*-1 in EC600, and the resensitization efficiency was over 99%. These findings indicate that when the ratio of donor-recipient bacteria was optimized, the effectiveness of the resensitization system increased significantly due to the increase in conjugation frequency, which allowed more recipient bacteria to acquire the CRISPR system. It is worth noting that 20 tigecycline-resistant and 13 colistin-resistant transconjugants harboring pRG and pTra/sgRNA simultaneously indicated that escape mutants may occur. After sequencing the resensitization system and *tet*(X4)/*mcr-1* targets of those escape isolates, it was observed that 30 out of 33 escape mutants exhibited base deletions within the sgRNA region, specifically the sequence near the crRNA. Additionally, three escape mutants were found to have deletions within the Cas9 promoter (Table S3).

### CRISPR/Cas system resensitizes clinical resistance plasmids

**Fig 3:**
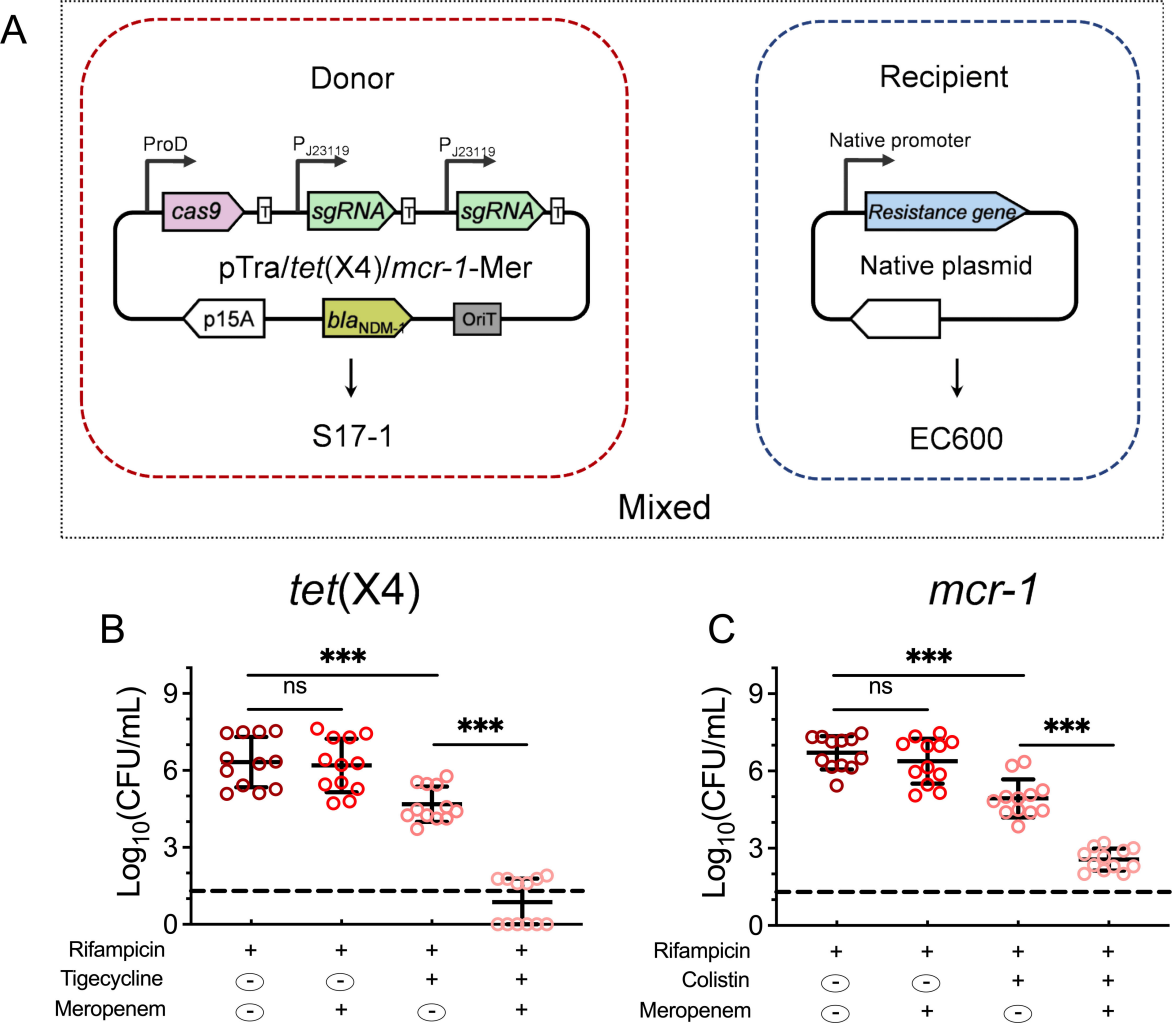
Engineered CRISPR-Cas9 system resensitizes clinical isolates through conjugation. (**A**) A schematic map of conjugative CRISPR/Cas9 system with meropenem-selected marker as the donor strain. Rifampicin-resistant *E. coli* EC600 carrying *tet*(X4) or *mcr-1* gene-positive native plasmid was set as the recipient strain. (**B and C**) Conjugative pTra/*tet*(X4)/*mcr-1-*Mer harboring two single-guide RNAs (sgRNAs) cause specific resensitivity to tigecycline or colistin. *E. coli* S17-1 donor cells containing pTra/*tet*(X4)/*mcr-1-*Mer were mated with *E. coli* EC600 recipient cells carrying different clinical plasmids with a donor-recipient ratio of 1:0.01 for 12h. The number of surviving colonies on the Luria–Bertani (LB) plate supplied with different antibiotics was calculated. Dashed lines indicate detection limitations (200CFU/mL). ND indicates that no clones have been observed on the plates. Data are shown as mean ± SD,and one‐way analysis of variance (ANOVA) was used to evaluate the statistical significance (ns, not significant; ***, *P* < 0.001).

MDR plasmids in clinical isolates usually contain several mobile genetic elements, such as insertion sequences, transposons, or integrative conjugative elements. To investigate the resensitization efficiency of MDR plasmids in clinical isolates, 12 clinical plasmids carrying *tet*(X4) or *mcr-1* were transformed into EC600 and were set as recipient strains separately (Fig. 3A). Since most of these clinical isolates were resistant to chloramphenicol but not to meropenem, the antibiotic marker of the resensitization system was replaced with a meropenem-resistant gene, *bla*_NDM-1_, so that the transconjugants could be screened using meropenem and rifampicin. Owing to the large number of resistance genes in the native plasmids, the sgRNA/*tet*(X4) and sgRNA/*mcr-1* were integrated into one resensitization system to resensitize tigecycline- and colistin-resistant strains simultaneously. After 12 h of conjugation, the CFU on rifampicin was comparable to that on the tigecycline or colistin plate, which indicates that the total number of recipients and transconjugants is the same (Fig. 3B and C). Additionally, the conjugation efficiency had reached 100%, and the resensitized efficiency was approximately 90%. Resistant transconjugants were significantly decreased in pTra/*tet*(X4)/*mcr-1*-Mer compared to the control group. In addition, when the resensitization system was applied to the chromosome-borne *mcr-1*-positive strain, no detectable clones were observed on the selected plate (data not shown). Overall, these findings demonstrate that we successfully constructed a CRISPR/Cas system that can resensitize clinical plasmid-mediated tigecycline- and colistin-resistant strains.

### CRISPR/Cas system resensitizes drug-resistant *E. coli in vivo*

**Fig 4:**
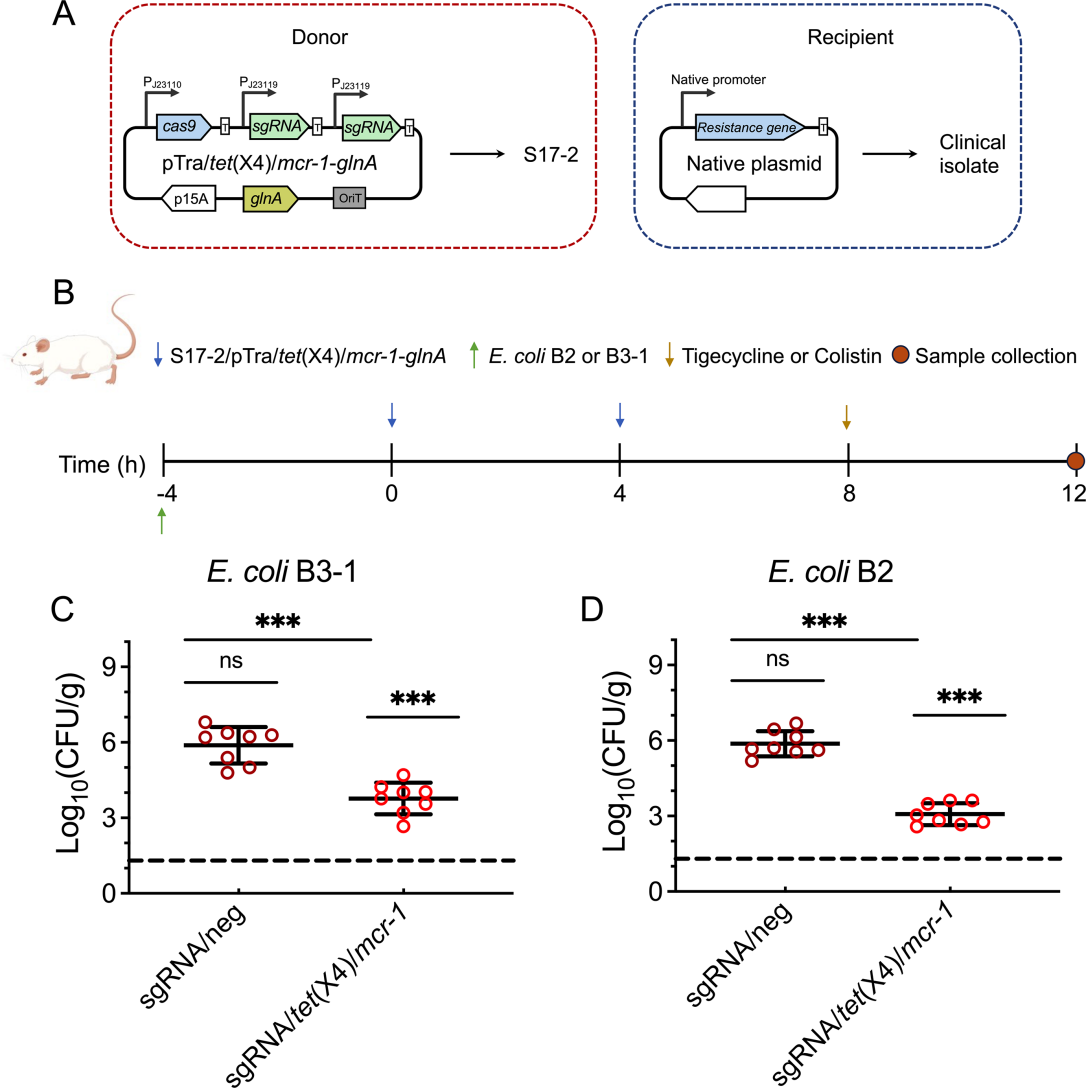
CRISPR/Cas9 system resensitizes clinical isolates *in vivo*. (**A**) A schematic map of a *glnA*-dependent vector–host system in S17-2 was set as the donor strain. Clinical isolates *mcr-1*-positive *E. coli* B2 or *tet*(X4)-positive *E. coli* B3-1 strains were set as the recipient strain. (**B**) Experimental design for testing the effect of resensitization in mice. (**C and D**) *E. coli* B2 or B3-1 load in the feces after colistin- or tigecycline-treated mice at 12h post-B2 or B3-1 oral administration (*n* = 8). Dashed lines indicate detection limitations (200CFU/mL). ND indicates that no clones have been observed on the plates. Data are shown as mean ± SD,and one‐way analysis of varince (ANOVA) was used to evaluate the statistical significance (ns, not significant; ***, *P* < 0.001).

To investigate the resensitization efficiency *in vivo*, we established an intestinal conjugation system. In order to assess the conjugation efficiency *in vivo*, varying quantities of S17-1-pTra/neg-Mer were gavaged 8 h after a 5 × 10^8^ or 5 × 10^9^ CFU of the *E. coli* B3-1 strains was administered. As shown in Fig. S4, the maximum conjugation efficiency *in vivo* is reached when the ratio of donor–acceptor bacteria was 10:1. Moreover, to maintain the CRISPR/Cas system with an obligatory requirement for glutamine synthetases since additional resistance genes cannot be introduced during *in vivo* experiments, a *glnA*-dependent vector-host system was developed. *E. coli* S17-1 without *glnA* (S17-2) required glutamine for growth, and for complementation, pTra/*glnA* was constructed by replacing the chloramphenicol-resistant gene with *glnA* under the native promoter in pTra/*tet*(X4)/*mcr-1*-Mer. Since the pTra/*glnA* lacks a resistant marker, the transconjugants cannot be identified and the survival-resistant bacteria were counted on LB plates supplied with tigecycline. As presented in Fig. 4, we observed that S17-2 harboring the resensitive system can significantly decrease the counts of B3-1 after being administered with tigecycline (*P* < 0.05), and the CFU on LB plates supplied with tigecycline is less than 1% compared to the negative control. Similar results were obtained when administered with colistin and *mcr-1*-positive clinical isolates of *E. coli* B2. These results indicate that the engineered conjugative CRISPR-Cas9 system can resensitize tigecycline- and colistin-resistant bacteria by targeting *tet*(X4) and *mcr-1* genes *in vivo*.

## DISCUSSION

Currently, the most effective antibiotics for treating Gram-negative bacterial infections that are resistant to carbapenems are tigecycline and colistin. However, the presence and spread of *tet*(X4) and *mcr-1* pose a significant obstacle to conventional clinical infection therapy. Therefore, developing new approaches to combat the spread of *mcr-1* and *tet*(X4), and restoring bacterial sensitivity to tigecycline and colistin is of significant scientific value.

The CRISPR/Cas9 method has been created to restore sensitivity in bacteria (10). A previous study developed an ISApl1-carried CRISPR/Cas9 system that can either eliminate plasmid-borne *mcr-1* or directly eradicate the bacteria-harboring chromosome-borne *mcr-1* (18). However, these methods were based on *in vitro* experiments, and the effectiveness of the treatment was focused on the percentage of resensitized clones among transconjugants. Since this method requires delivery of the resensitized system by conjugation, the percentage of conjugated (successfully delivered) bacteria in the total bacterial population is critical to the resensitized efficiency. Furthermore, the conjugation efficiency was relatively lower in clinical isolates, and the content of the plasmid backbone influenced efficiency. In a universal plasmid-curing system with elimination efficiencies up to 100% in cloning vector (14), the elimination rate in natural resistance plasmid is only 18/24 (19). This study presents a strategy that increases the ratio between donor and recipient by over 1:0.01, which allows almost 100% of the individuals in a bacterial population to be successfully conjugated. After conjugating into the target bacteria, the CRISPR-Cas9 system introduced double-stranded breaks in the target DNA. The plasmid-borne *tet*(X4)- or *mcr-1*-positive bacteria were eliminated when treated with a low dose of tigecycline or colistin. Owing to the high efficiency of conjugation, less than 1% of the bacteria still harbor *tet*(X4) or *mcr-1* and survive after antibiotic treatment. Furthermore, there was no detectable co-existence of the resistant gene, and the resensitized system indicated that the escape rate is relatively low.

Although the CRISPR resensitized system delivered by transconjugation has demonstrated its effectiveness as a potent programmable antimicrobial, there are several limitations of this delivery system. First, the primary obstacle in eradicating the bacteria responsible for infectious diseases is achieving an efficient delivery in clinically relevant settings. The inter-species transconjugation frequency is often lower than the intra-species transconjugation frequency. For example, the *E. coli* carrying the resensitization system as the donor is often less efficient when attempting to target other species, especially Gram-positive bacteria (20), and the conjugation efficiency is also diminished when the target bacteria are clinical isolates. The present study proposed a way to achieve a greater donor-to-recipient ratio that enables a nearly 100% conjugation rate among individuals in a bacterial community. However, the relationship between specificity and efficiency seems contradictory, as a high delivery efficiency leads to low specificity due to the capability of the resensitized system to transfer into both pathogens and probiotic bacteria. In addition, the remaining plasmid in probiotic bacteria might potentially serve as a source of gene contamination. Last but not least, transconjugation is specifically effective in eradicating pathogenic bacteria in the gastrointestinal tract, but it does not have any impact on bacterial infections in other organs inside the body. Indeed, a delivery format is anticipated to vary depending on the target organism and the site of infection (i.e., intestinal vs topical vs lung infections vs urogenital tract). Nowadays, the progress in phage technology and nanotechnology may provide new ideas for the delivery of CRISPR-Cas systems to expand the safety and effective application of antimicrobial therapies.

It is worth noting that the conjugative platforms have been employed for the eradication of infections, antimicrobial resistance (AMR) genetic determinants, or the bacteria harboring them (16, 21–23). However, efficient elimination *in vivo* has received less attention in earlier studies. In this research, the resensitized system transconjugated with and resensitized the *tet*(X4)-mediated tigecycline-resistant strains and *mcr-1-*mediated colistin-resistant strains *in vivo*. The results revealed that the resensitization system can destroy *tet*(X4)- and *mcr-1*-resistant genes, which helps antibiotics eliminate the infected bacterium in the gut. It is a challenge when designing an experiment to eliminate a drug-resistant gene while simultaneously introducing another resistant gene. Therefore, the resistant gene that is always used as a selected marker to maintain plasmid stability is no longer viable *in vivo*. The auxotroph bacteria cannot survive without an exogenous nutrient supplement, but when a complementary plasmid was introduced, the growth rate and viability were comparable to those of the wild-type strain. This system replaces the antibiotic resistance marker with a nutrient selection marker, allowing a stable expression of the exogenous gene in the host bacterium (24). In this research, we exploited this essential requirement for glutamine biosynthesis to develop a *glnA*-dependent vector–host system in *E. coli* S17-1. In addition to preventing the introduction of new resistant genes, the chromosome-plasmid-balanced lethal system also provides a method of strain biocontainment, where the resensitization system may promote their clearance after executing their programmed function or when the expression construct is lost (25).

To the authors’ knowledge, this is the first research on simultaneously resensitizing *mcr-1-* and *tet*(X4)-mediated resistant isolates *in vivo*. This system replaced the antibiotic-resistant marker with a chromosome-plasmid-balanced lethal system, which avoids the spread of the antibiotic-resistant trait of the plasmid. It serves as a template for a strategy that might be used to combat the deterioration of tigecycline and colistin resistance among bacterial infections. Future research will focus on designing broad-targeted sgRNA on different resistant genes and screening plasmids with high conjugation efficiency in broad-range host strains. In addition, developing probiotics as conjugated host systems will help to further improve this resensitized system. Conclusively, given the fact that bacterial conjugation is a ubiquitous and natural process among bacteria, the conjugative CRISPR/Cas9 system could be a favorable therapeutic application in a clinical setting and could have the potential to eliminate antibiotic resistance genes in the environment.

## MATERIALS AND METHODS

### Bacterial strains, plasmids, and growth conditions

The bacterial strains and plasmids used or generated in this study are listed in Table S1. Cloning experiments were performed in *E. coli* DH5α, and LB was used as the cloning medium unless otherwise stated. *E. coli* B3-1 and B2 used *in vivo* experiments were both infected bacteria that were isolated from diarrheic piglets. The antibiotics, ampicillin (100 µg/mL), chloramphenicol (25 µg/mL), kanamycin (50 µg/mL), rifampicin (200 µg/mL), tigecycline (2 µg/mL), colistin (2 µg/mL), and meropenem (2 µg/ mL), were added into the medium for selection when required. Glutamine (25 µg/mL) was supplied to maintain the auxotrophic strain’s growth. All strains were cultured at 37°C.

### Generation of glutamine auxotrophic strain

The glutamine synthetase gene *glnA* gene of *E. coli* S17-1 was deleted using the pRE112 suicide plasmid to generate a glutamine auxotrophic strain, S17-2 (26). Briefly, the upstream and downstream homologous arms of *glnA* were amplified using *glnA*-up-F/R and *glnA*-down-F/R. Both fragments were purified and fused in an overlap-extension PCR using primers *glnA*-up-F and *glnA*-down-R. The fused product was ligated into the *Xba*I/*Xma*I sites of pRE112 and was transformed into *E. coli x7213*. The plasmid pRE112-Δ*glnA* was conjugated into S17-1, and eight transconjugants were randomly selected from the LB plate containing chloramphenicol. The second exchange clone was counter-selected on LB plate containing 10% sucrose to ensure the excision of pRE112 from the chromosome. The auxotrophic candidates were tested for both the deletion of the *glnA* gene by PCR and the growth conditions with or without glutamine.

### sgRNA design and plasmid construction

First, p15A Ori and chloramphenicol-resistant markers were amplified with primers p15A-F/cmR-R from pdCas9-bacteria plasmid and were ligated with *Afl*II-*Arv*II digested plasmid pwtCas9-bacteria to generate the chloramphenicol-resistant low-copy number plasmid pCas/Ind. At the same time, *Xma*I, *Asi*SI, and *Not*I were introduced into pCas/Ind for a more convenient subsequent plasmid construction. Strong insulated promoter proD (17) and ribosome binding site (AAAGAGGAGAAA) were replaced with aTc-induced *tet*A promoter in pCas/Con. Furthermore, to assemble pTran/Ind and pTran/Con, an origin of transfer (oriT) and sgRNA components were amplified with primers oriT-F/R and sgRNA-F/R from plasmid pRE112 and psgRNA, respectively. Both fragments were purified and fused in an overlap-extension PCR using primers oriT-F and sgRNA-R, ligated with *BsrB*I digested plasmid pCas/Ind or pCas/Con. To construct a plasmid harboring different sgRNAs, a 20-nucleotide base-pairing region (N20) of an sgRNA was designed using the CHOPCHOP tool (http://chopchop.cbu.uib.no/) and was amplified with primers N20-GTTTTAGAGCTAGAAATAGC and N20_(reverse complement)_-ACTAGTATTATACCTAGGAC using psgRNA as the template. The PCR products were purified by cycle-pure kit (OMEGA, China) and digested with *Dpn*I at 37°C to remove the template DNA. The digested products were transformed into DH5α competent cells and subjected to DNA sequencing. To construct pTran/sgRNA, each module with different sgRNAs was amplified using sgRNA-F/R and was ligated into *Xma*I/*Asi*SI digested pTran/Con. To construct pTran/*tet*(X4)/*mcr-1*-Mer and pTran/*tet*(X4)/*mcr-1-glnA*, *bla*_NDM-1_ and *glnA* with their promoters were amplified using *bla*_NDM-1_-F/R and *glnA*-F/R, respectively, replacing the chloramphenicol-selected marker. All sgRNA and primers used in this study are listed in Table S2.

### Plasmid transformation assays

For the transformation assay, the competent bacteria containing *mcr-1* and *tet*(X4) dual expression system were prepared following the protocol of Chung et al. (27). A 100 µL aliquot of cells mixed with 100 ng of pCas/Con and 50 ng of psgRNA harboring different sgRNAs were incubated on ice for 30 min and subsequently heat shocked at 42°C for 60 s. Then, 0.9 mL of super optimal broth (SOB) was added to the mixture, which was then shaken while being incubated at 37°C for 60 min. Serial dilutions of cells were spread on LB agar plates supplied with different antibiotics and inducers. After overnight incubation at 37°C, transformation frequencies (expressed as colony forming unit [CFU] per microgram of DNA transformed) were calculated.

### Bacterial growth tests

Three independent clones were randomly selected from DH5α containing *mcr-1* and *tet*(X4), co-transformed with pCas/Ind and psgRNA harboring different sgRNAs, and inoculated in 96 microplates containing LB media supplemented with various antibiotics. The growth curves were determined using Infinite E Plex microplate reader (Tecan) with absorbance (620 nm) recorded at a 20-min interval for a period of 8 h.

### Resensitizing tigecycline- and colistin-resistant *E. coli in vitro*

Conjugation assays were performed using S17-1 harboring pTran/*tet*(X4)/*mcr-1* as the donor strain and rifampicin-resistant *E. coli* EC600 transformed with *mcr-1* and *tet*(X4) as the recipient strain. Briefly, the overnight-grown donor and recipient strains were diluted at a ratio of 1:100 in fresh medium and were incubated to reach an OD_600_ of 0.5. After being washed with phosphate-buffered saline (PBS), the receiver was diluted at various multiples. The donor and recipient were then mixed at a ratio ranging from 1:1 to 1:0.0001, and 50 µL of the mixture was applied onto GN-6 membrane (Pall) above LB agar plates. After settling at 37°C for 12  h, the GN-6 membrane was carefully removed and transferred separately into 1 mL sterile PBS. The mixtures were thoroughly vortexed, serially diluted in PBS, and counted on an LB plate supplied with different antibiotics. The transconjugation efficiency was calculated by the CFU of transconjugants (CFU/mL) per number of recipients (CFU/mL). All experiments were performed with at least three biological replicates.

### Resensitizing tigecycline- and colistin-resistant *E. coli in vivo*

Female BALB/c mice that were 6–8 weeks old were procured from Yangzhou University’s Comparative Medicine Centre (Jiangsu, China). The mice were allowed to adapt to the environment for a week before being infected. Mouse studies were performed per the relevant guidelines and regulations (ID: SYXK-2021-0026), certified by Jiangsu Association for Science and Technology. The mice were treated with antibiotics (1 g/L streptomycin, 0.5 g/L ampicillin, 1 g/L gentamicin, and 0.5 g/L vancomycin) to clear the gut microbiota. Conjugation assays *in vivo* were performed using clinical isolate *mcr-1*-positive *E. coli* B2 or *tet*(X4)-positive *E. coli* B3-1 as the recipient strain. Briefly, oral administration of donor strain at 5 × 10^8^ or 5 × 10^9^ CFU was given to 6- to 8-week-old female ICR mice (*n* = 8 per group) 4 h or 8 h after 5× 10^8^ CFU B2 or G93, respectively. In resensitizing experiments, tigecycline or colistin was administered by oral injection at 20 mg/kg 4 h later. To monitor colonization, fecal samples were collected, weighed, and then diluted with PBS. LB plates treated with appropriate antibiotics were used to count the number of CFUs.

## SUPPLEMENTAL MATERIAL

Tables S1 to S3, Fig. S1 to S4 spectrum.03884-23-s0001.docx Strains, primers, and additional figures.

ASM does not own the copyrights to Supplemental Material that may be linked to, or accessed through, an article. The authors have granted ASM a non-exclusive, world-wide license to publish the Supplemental Material files. Please contact the corresponding author directly for reuse.
