# Supplementary material for: Resensitizing tigecycline- and colistin-resistant Escherichia coli using an engineered conjugative CRISPR/Cas9 system
Source: Microbiol Spectr. 2024 Feb 22;12(4):e03884-23. doi: 10.1128/spectrum.03884-23 (PMC10986596; doi:10.1128/spectrum.03884-23)
Supplement: Tables S1 to S3, Fig. S1 to S4 — Strains, primers, and additional figures. [file spectrum.03884-23-s0001.docx]

**Supplementary Table 1. Strains and plasmids used in this study**

| Strain or Plasmid | Relevant genotype or phenotype | Reference |
| --- | --- | --- |
| Strain |  |  |
| *E. coli* DH5α | *F^-^ φ80 lacZΔM15 Δ(lacZYA-argF) U169 recA1 endA1 hsdR17(rk^-^, mk^+^) phoA, supE44 thi-1 gyrA96* | Laboratory stock |
| *E. coli* MG1655 | *F^-^ λ ilvG rfb-50 rph-1* | Laboratory stock |
| *E. coli* S17-1 | *RP4-2(Km::Tn7,Tc::Mu-1) pro-82 LAMpir recA1 endA1 thiE1 hsdR17 creC510* | Laboratory stock |
| *E. coli* EC600 | (Rif^R^) | Laboratory stock |
| *E. coli* *x7213* | thi-1 thr-1 leuB6 fhuA21 lacY1 glnV44 asdA4 recA1 RP4 2-Tc::Mu pir; Km^r^ | Laboratory stock |
| *E. coli* S17-2 | *E. coli* S17-1 Δ*glnA* | This study |
| *E. coli* B2 | *mcr-1-*positive clinical *E. coli* isolate | [1] |
| *E. coli* B3-1 | *tet*(X4)*-*positive clinical *E. coli* isolate | [2] |
| Plasmid |  |  |
| pRE112 | Widely used gene-knocked suicide vector with oriT RP4 and R6K γ ori, (Cm^R^) | Laboratory stock |
| pRE112-Δ*glnA* | pRE112 containing the homologous arms of *glnA*, (Cm^R^) | This study |
| pRG | *mcr-1* and *tet*(X4) dual expression system, pSC101, (Amp^R^) | This study |
| psgRNA | sgRNA expression vector, ColE1, (Kan^R^) | Addgene #114005 |
| pdCas9-bacteria | aTc-inducible expression of a dCas9, p15A, (Cm^R^) | Addgene #44249 |
| pwtCas9-bacteria | aTc-inducible expression of a Cas9, ColE1, (Amp^R^) | Addgene #44250 |
| pCas/Ind | aTc-inducible expression of a Cas9, p15A, (Cm^R^) | This study |
| pCas/Con | Constitutive promoter J23110 and RBS in pCL011 | This study |
| pTra/Ind | oriT and sgRNA/neg in pCas/Ind | This study |
| pTra/Con | oriT and sgRNA/neg in pCas/Con | This study |
| pTra/*tet*(X4) | sgRNA/*tet*(X4)-1 in pTra/Con | This study |
| pTra/*mcr-1* | sgRNA/*mcr-1*-1 in pTra/Con | This study |
| pTra/*tet*(X4)/*mcr-1* | sgRNA/*tet*(X4)-1 and sgRNA/*mcr-1*-1 in pTra/Con | This study |
| pTra/neg-Mer | *bla*_NDM-1_ in pTra/Con, (Mer^R^) | This study |
| pTra/*tet*(X4)/*mcr-1*-Mer | *bla*_NDM-1_ in pTra/*tet*(X4)/*mcr-1*, (Mer^R^) | This study |
| pTra/*tet*(X4)/*mcr-1*-*glnA* | *glnA* in pTra/*tet*(X4)/*mcr-1*, (*glnA*) | This study |
| psgRNA-*tet*(X4)-1 | sgRNA/*tet*(X4)-1 in psgRNA | This study |
| psgRNA-*tet*(X4)-2 | sgRNA/*tet*(X4)-2 in psgRNA | This study |
| psgRNA-*tet*(X4)-3 | sgRNA/*tet*(X4)-3 in psgRNA | This study |
| psgRNA-*mcr-1*-1 | sgRNA/*mcr-1*-1 in psgRNA | This study |
| psgRNA-*mcr-1*-2 | sgRNA/*mcr-1*-2 in psgRNA | This study |
| psgRNA-*mcr-1*-3 | sgRNA/*mcr-1*-3 in psgRNA | This study |

Ap, ampicillin; Cm, chloramphenicol; Km, kanamycin; Rif, rifampicin; Mer, meropenem; *glnA*, glutamine synthetase gene.

[1] Fang D., Xu T., Sun J., Shi J., Li F., Yin Y., Wang Z., and Liu Y. Nicotinamide mononucleotide ameliorates sleep deprivation-induced gut microbiota dysbiosis and restores colonization resistance against intestinal infections. Advanced Science, 2023. 10(9): e2207170.

[2] Liu Y., Jia Y., Yang K., Li R., Xiao X., and Wang Z. Anti-HIV agent azidothymidine decreases Tet(X)-mediated bacterial resistance to tigecycline in *Escherichia coli*. Communications Biology, 2020. 3(1):162.

**Supplementary Table 2. Oligonucleotides used in this study**

| Primers and sgRNAs | Sequence |
| --- | --- |
| Primers |  |
| *glnA*-up-F | TCCCAAGCTTCTTCTAGATGTTGGAGCAGCTTGTCT |
| *glnA*-up-R | CGGCAACTAAAACACTTACACTTCGTGCTCGTTCAG |
| *glnA*-down-F | CTGAACGAGCACGAAGTGTAAGTGTTTTAGTTGCCG |
| *glnA*-down-R | ACTGCATGAATTCCCGGGTCCAGACTTTCTTGCATC |
| oriT-F | GTTCGTAAGCCATTTCCGGCGGCCGCTTTCCGCTGCATAACCCT |
| oriT-R | TTATCACTTTACGGGTCCGCGATCGCGACCCAGGCGCTCGGTCT |
| sgRNA-F | AGACCGAGCGCCTGGGTCGCGATCGCGGACCCGTAAAGTGATAA |
| sgRNA-R | GTCGTTCGACTGCGGCGAGGCGATCCCGGGATTAAGTTCTGTGCTAGG |
| p15A-F | TCTGCGTTTATACCTAGGGATATATTCCGCTTCCTCG |
| cmR-R | AGTGGGTCTTAAGACGTCGATATCTGGCGAAAATGAGA |
| *bla*_NDM-1_-F | AGCGAGCTCGATATCAAAGTCGACTCAGCGCAGCTTGTCGGC |
| *bla*_NDM-1_-R | AGTGGGTCTTAAGACGTCGATGAACCCTGTTCCATG |
| *glnA*-F | AGCGAGCTCGATATCAAATTAGACGCTGTAGTACAGC |
| *glnA*-R | AGTGGGTCTTAAGACGTCAAGTATTGCAGAGTCCCT |
| sgRNAs |  |
| sgRNA/neg | AACTTTCAGTTTAGCGGTCT |
| sgRNA/*tet*(X4)-1 | CTGAACCTTTGTGTAGGTCA |
| sgRNA/*tet*(X4)-2 | CGAAATTAATTACTCTTGAA |
| sgRNA/*tet*(X4)-3 | TTGTTTAACTTTAAGAAGGAGAT |
| sgRNA/*mcr-1*-1 | TTTATCAAAAAAGGTAAGAT |
| sgRNA/*mcr-1*-2 | GGCGGTCGCGGTCAAGAAAA |
| sgRNA/*mcr-1*-3 | AAAGGTAAGATTGGCGGTCG |

**Supplementary Table 3. Sequence information of escape mutants that tolerate the resensitization system.**

|  | Sequence | Counts | |
| --- | --- | --- | --- |
| Original sequence  of sgRNA | TTGACAGCTAGCTCAGTCCTAGGTATAATACTAGTCTGAACCTTTGTGTAGGTCAGTTTTAGAGCTAGAAATAGCAAGTTAAAATAAGGCTAGTCCGTTATCAACTTGAAAAAGTGGCACCGAGTCGGTGCTTTTTTT |  | |
| Mutations in sgRNA | TTGACAGCTAGCTCAGTCCTAGGTATAATACTA~~GT~~CTGAACCTTTGTGTAGGTCAGTTTTAGAGCTAGAAATAGCAAGTTAAAATAAGGCTAGTCCGTTATCAACTTGAAAAAGTGGCACCGAGTCGGTGCTTTTTTT | *tet*(X4):  11/20 | *mcr-1*:  3/13 |
|  | TTGACAGCTAGCTCAGTCCTAGGTATAATACTAG~~T~~CTGAACCTTTGTGTAGGTCAGTTTTAGAGCTAGAAATAGCAAGTTAAAATAAGGCTAGTCCGTTATCAACTTGAAAAAGTGGCACCGAGTCGGTGCTTTTTTT | *tet*(X4):  5/20 | *mcr-1*:  5/13 |
|  | TTGACAGCTAGCTCAGTCCTAGGTATAATACTAGTCTGAACCTTTGTGTAGGTCA~~GTTTT~~AGAGCTAGAAATAGCAAGTTAAAATAAGGCTAGTCCGTTATCAACTTGAAAAAGTGGCACCGAGTCGGTGCTTTTTTT | *tet*(X4):  1/20 | *mcr-1*:  0/13 |
|  | TTGACAGCTAGCTCAGTCCTAGGTATAATACTAGTCTGAACCTTTGTGTAGGTCA~~G~~TTTTAGAGCTAGAAATAGCAAGTTAAAATAAGGCTAGTCCGTTATCAACTTGAAAAAGTGGCACCGAGTCGGTGCTTTTTTT | *tet*(X4):  0/20 | *mcr-1*:  1/13 |
|  | TTGACAGCTAGCTCAGTCCTAGGTATAATACTAGTCTGAACCTTTGTGTAGGTCA~~GT~~TTTAGAGCTAGAAATAGCAAGTTAAAATAAGGCTAGTCCGTTATCAACTTGAAAAAGTGGCACCGAGTCGGTGCTTTTTTT | *tet*(X4):  1/20 | *mcr-1*:  4/13 |
| Original sequence  Cas9 promoter | TTCTAGAGCACAGCTAACACCACGTCGTCCCTATCTGCTGCCCTAGGTCTATGAGTGGTTGCTGGATAACTTTACGGGCATGCATAAGGCTCGTATAATATATTCAGGGAGACCACAACGGTTTCCCTCTACAAATAATTTTGTTTAACTTTTACTAGAGAAAGAGGAGAAAGGATCT |  |  |
| Mutations in  Cas9 promoter | TTCTAGAGCACAGCTAACACCACGTCGTCCCTATCTGCTGCCCTAGGTCTATGAGTGGTTGCTGGATAACTTTACGGGCATGCATAAGGCTCGTATAATATATTCAGGGAGACCACAACGGTTTCCCTCTACAAATAATTTTGTTTAACTTTTACTAGAGAAA~~GAGGAG~~AAAGGATCT | *tet*(X4):  3/20 | *mcr-1*:  0/13 |

The highlight indicated the deletions at the sgRNA or Cas9 part which led to the resensitization system inactivation in the successful transconjugants.

**Supplementary Figures**


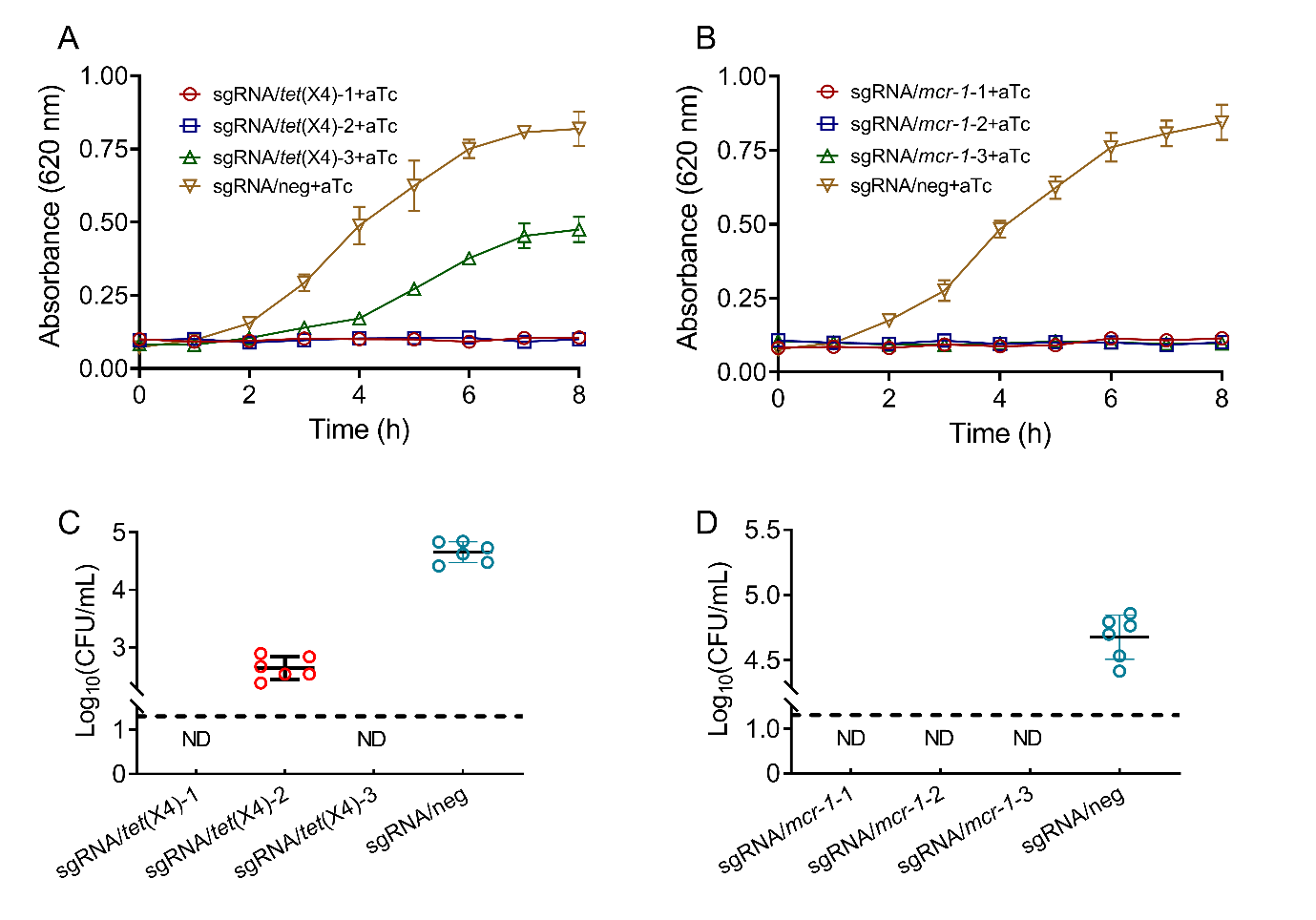


**Supplementary Figure 1** Clustered regularly interspaced short palindromic repeats (CRISPR)-associated protein 9 (Cas9) system resensitized *tet*(X4)- and *mcr-1*-mediated tigecycline- and colistin-resistant *E. coli*. **(A)** Growth curve of DH5α transformed with pRG, pCas/Ind, and psgRNA carrying sgRNA/*tet*(X4)-1, sgRNA/*tet*(X4)-2, sgRNA/*tet*(X4)-3 or sgRNA/neg, **(B)** sgRNA/*mcr-1*-1, sgRNA/*mcr-1*-2, sgRNA/*mcr-1*-3 or sgRNA/neg with 1 μM aTc treatment. **(C)** The number of surviving colonies on the LB plate supplied with ampicillin (100 μg/mL), chloramphenicol (25 μg/mL), and kanamycin (50 μg/mL) after transformation with pRG, pCas/Con, and psgRNA carrying sgRNA/*tet*(X4)-1, sgRNA/*tet*(X4)-2, sgRNA/*tet*(X4)-3 or sgRNA/neg, **(D)**sgRNA/*mcr-1*-1, sgRNA/*mcr-1*-2, sgRNA/*mcr-1*-3 or sgRNA/neg. Dashed lines indicate detection limitations (200 CFU/mL). ND indicates that no clones have been observed on the plates.


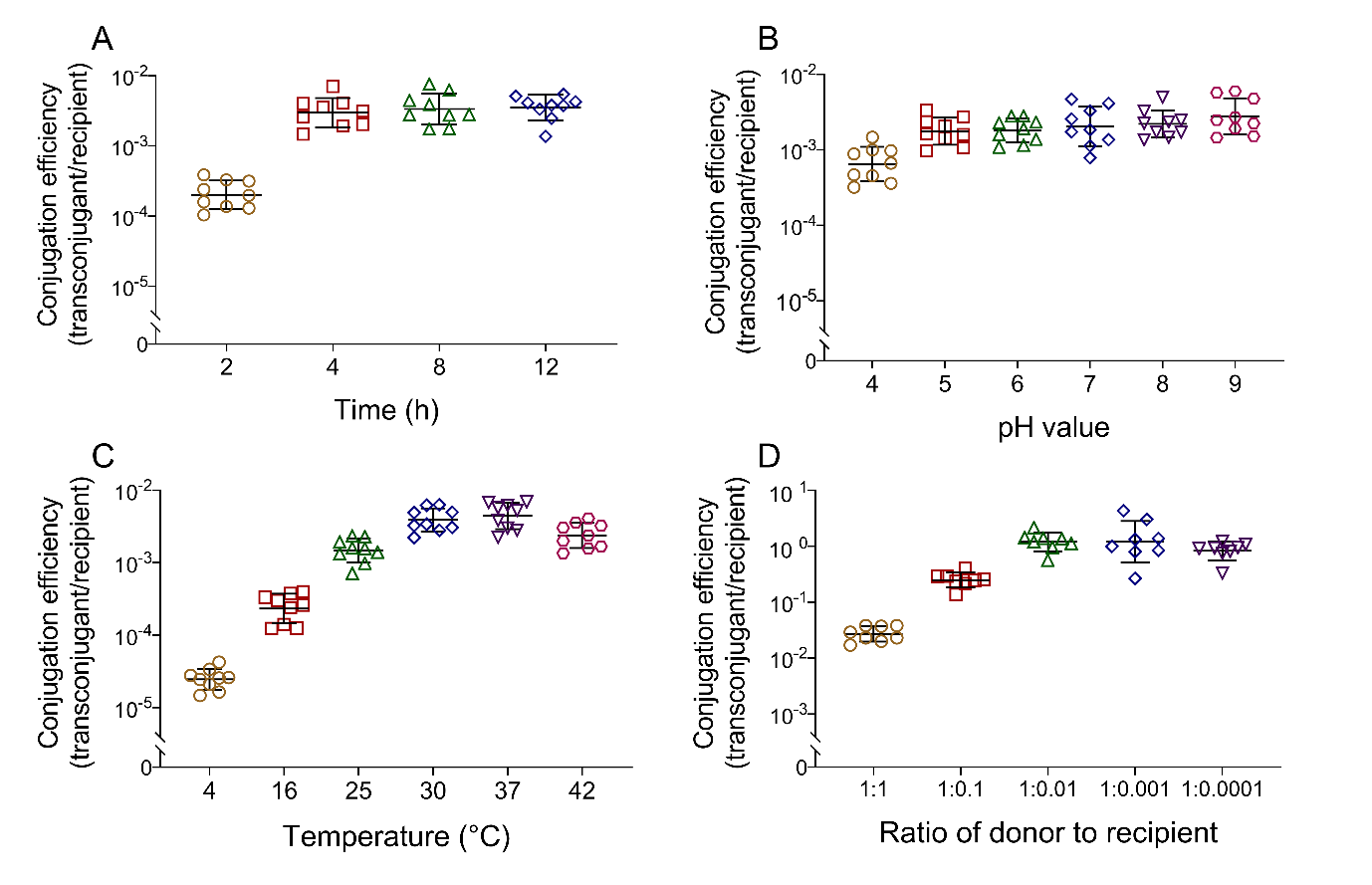


**Supplementary Figure 2** The conjugation efficiency of the resensitive system with **(A)** different mating times, **(B)** pH values, **(C)** temperatures, and **(D)** the donor-to-recipient ratio. The conjugation efficiency is measured as the ratio of transconjugant to recipient.


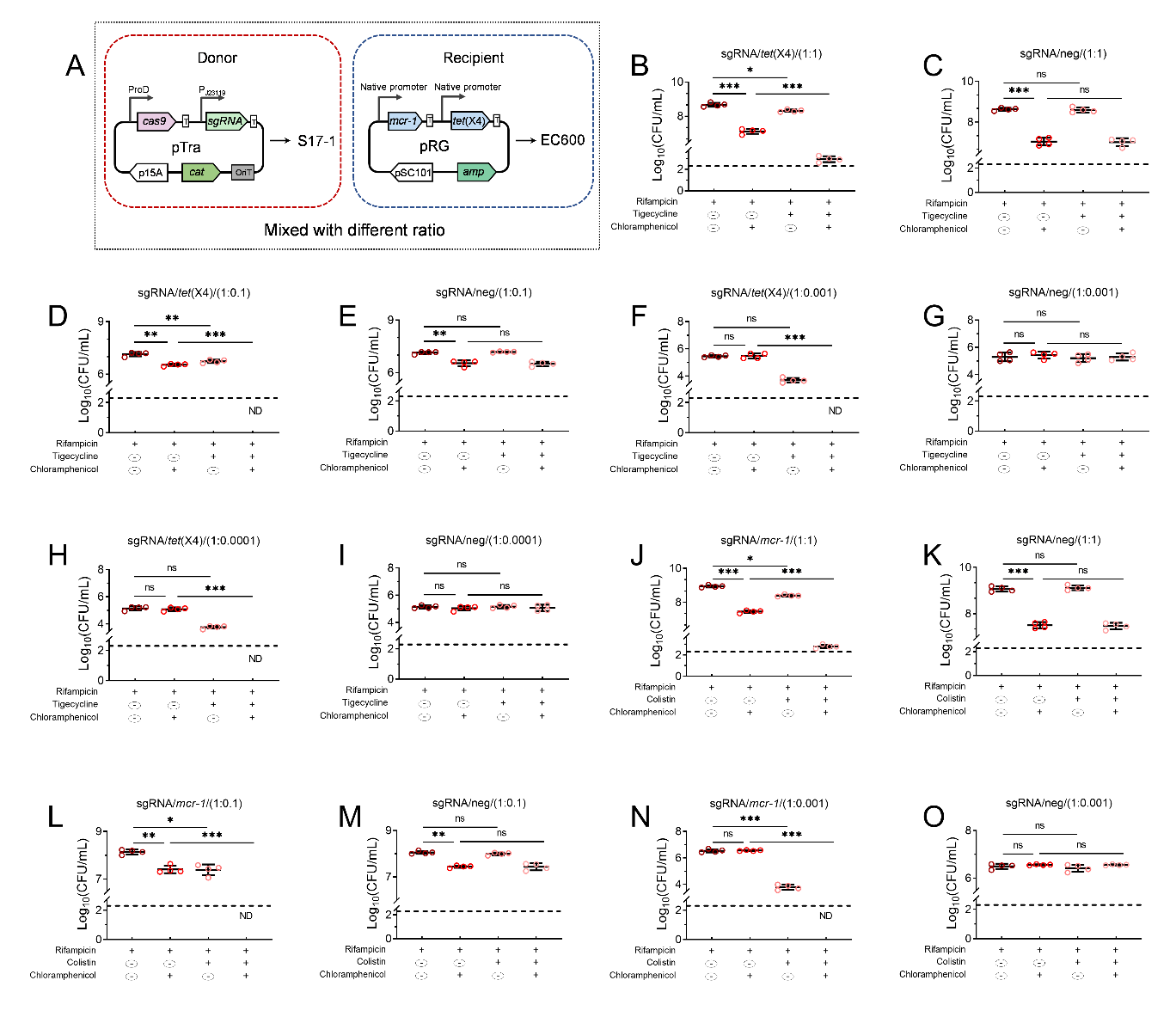


**Supplementary Figure 3** Clustered regularly interspaced short palindromic repeats (CRISPR)-associated protein 9 (Cas9) system can be delivered into target bacteria harboring target sequences by conjugation. **(A)** Schematic map of conjugative resensitization system. Conjugative CRISPR/Cas9 system harboring sgRNAs/*tet*(X4) or sgRNA/*mcr-1* cause specific clearance of plasmid pRG. *E. coli* S17-1 donor cells containing pTra/*tet*(X4) were mated with *E. coli* EC600 recipient cells carrying pRG at a donor:recipient ratio of 1:1 **(B and C)**, 1:0.1 **(D and E)**, 1:0.001 **(F and G)** and 1:0.0001 **(H and I)** for 12 h. S17-1 with pTra/*mcr-1* was mated with EC600 carrying pRG at a donor-recipient ratio of 1:1 **(J and K)**, 1:0.1 **(L and M),** and 1:0.001 **(N and O)**. Dashed lines indicate detection limitations (200 CFU/mL). ND indicates that no clones have been observed on the plates. Data were shown as mean ± SD and one‐way ANOVA was used to evaluate the statistical significance (ns, not significant, * *P* < 0.05, ** *P* < 0.01, *** *P* < 0.001).


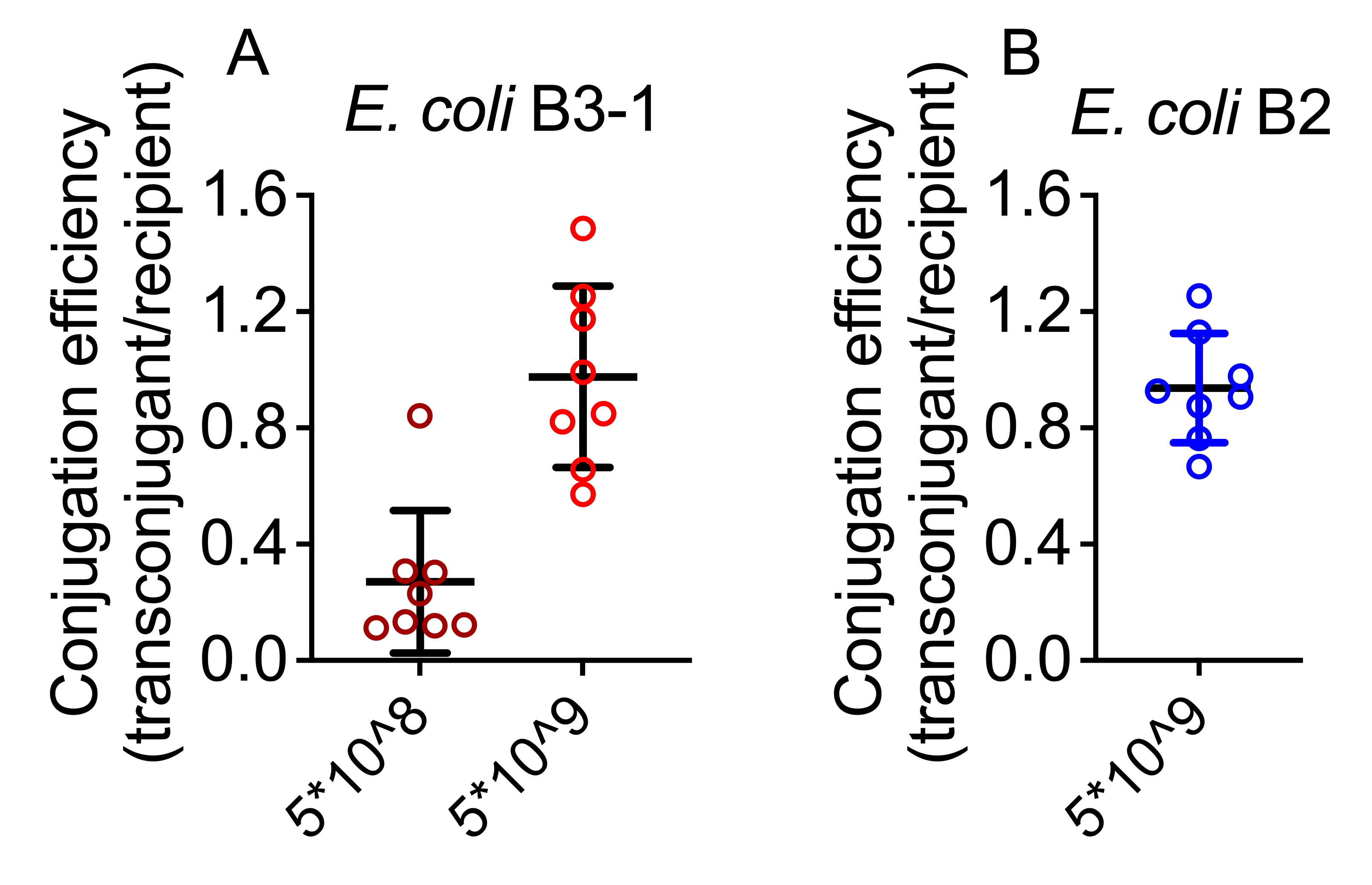


**Supplementary Figure 4** The conjugation efficiency of the resensitive system in vivo. *E. coli* S17-1 carrying pTra/neg-Mer mated with *E. coli* B3-1 **(A)** or B2 **(B)**. The conjugation efficiency is calculated as the ratio of transconjugant and recipient bacteria.
